# Crystal Structures and Antifungal Activities of Fluorine-Containing Thioureido Complexes with Nickel(II)

**DOI:** 10.3390/molecules181215737

**Published:** 2013-12-17

**Authors:** Chun Li, Wen Yang, Huanhuan Liu, Mengying Li, Weiqun Zhou, Juan Xie

**Affiliations:** 1 College of Pharmacy, Nantong Tichen Health School, Nantong 226007, China; 2 College of Chemistry, Chemical Engineering and Materials Science, Soochow University, 199 Ren’ai Road, Suzhou 215123, China; 3 School of Biology & Basic Medical Sciences, Soochow University, 199 Ren’ai Road, Suzhou 215123, China

**Keywords:** fluorobenzoyl thioureas, Ni(II) complexes, crystal structures, antifungal activity

## Abstract

Ni(II) complexes with *N*-2-fluorobenzoylpiperidine-1-carbothioimidate (L2^−^), *N*-4-fluorobenzoylpiperidine-1-carbothioimidate (L3^−^), N-2-fluorobenzoylmorpholine- 1-carbothioimidate (L5^−^) and N-4-fluorobenzoylmorpholine-1-carbothioimidate (L6^−^) have been synthesized and characterized by elemental analysis, FTIR and ^1^H-NMR. The crystal structures of three ligands (HL2, HL3 and HL6) and the corresponding Ni(II) complexes ([Ni(L2)_2_], [Ni(L3)_2_] and [Ni(L6)_2_]) have been determined by X-ray diffraction. The antifungal activities of the Ni(II) complexes together and the corresponding ligands against the fungi *Botrytis cinerea*, *Trichoderma* spp., *Myrothecium* and *Verticillium* spp. have been investigated. The experimental results showed that the ligands and their complexes have antifungal abilities. When the fluorine was substituted on the *para*-benzoyl moiety, the antifungal activity of the ligands was obviously increased. Moreover, the ligands were stronger than their complexes in inhibiting fungal activities. The antifungal ability of HL6 is especially strong, and similar to that of the commercial fungicide fluconazole.

## 1. Introduction

As we know, pyridine, morpholine and thiourea exhibit broad biological activities, including antibacterial and antifungal effects [1,2,3,4,5,6,7,8,9,10,11]. 1,3-Dialkyl- or diarylthioureas present significant antifungal activity against the plant pathogens *Pyricularia oryzae* and *Drechslera oryzae* [12]. Acyl thioureas are well known for their superior pesticidal, fungicidal, antiviral and plant growth regulating activity [13]. Moreover, thioureas containing both carbonyl and thiocarbonyl groups are versatile ambidentate donor ligands for transition metal ions [14].

The introduction of fluorine at a strategic position of a molecule is a powerful and versatile tool for the development of organic molecules which have potential biological activities by changing the steric and electronic parameters [15,16,17,18,19,20,21]. The inclusion of fluorine into organic molecules can increase the lipophilicity and thus enhance the rate of cell penetration and transport of a drug to an active site [22]. Fluorinated thioureas constitute a novel class of potent influenza virus neuraminidase inhibitors.

The biological activities of metal complexes are different from those of either their ligands or the free metal ions. In many cases, upon coordination to metal ions, the bioactivity of these complexes increases, suggesting that complexation can be an interesting dose reduction strategy [23,24,25,26,27,28]. However, some Co(III) and Ni(II) complexes had the weaker antibacterial activities than the corresponding ligands [11,29]. The proximate experiments of our group on fluorobenzoylthioureas and their Co(III) complexes [29] have revealed the introducing a fluorine atom on *para*-benzoylthiourea increased the antibacterial activities, while when the fluorine atom was substituted on an *ortho*-benzoyl group, the antibacterial activity was weakened.

In this paper, we synthesized four complexes, [Ni(L2)_2_], [Ni(L3)_2_], [Ni(L5)_2_] and [Ni(L6)_2_]. The crystal structures of the three ligands (HL2, HL3, HL6) and the corresponding complexes were determined by X-ray diffraction. In consideration of the knwon good biological effects of nickel ion and its complexes [30,31], we were interested in exploring the effect of fluorine substitution and the coordination with Ni (II) on the antifungal activities.

## 2. Results and Discussion

### 2.1. Structural Characteristic

**Figure 1:**
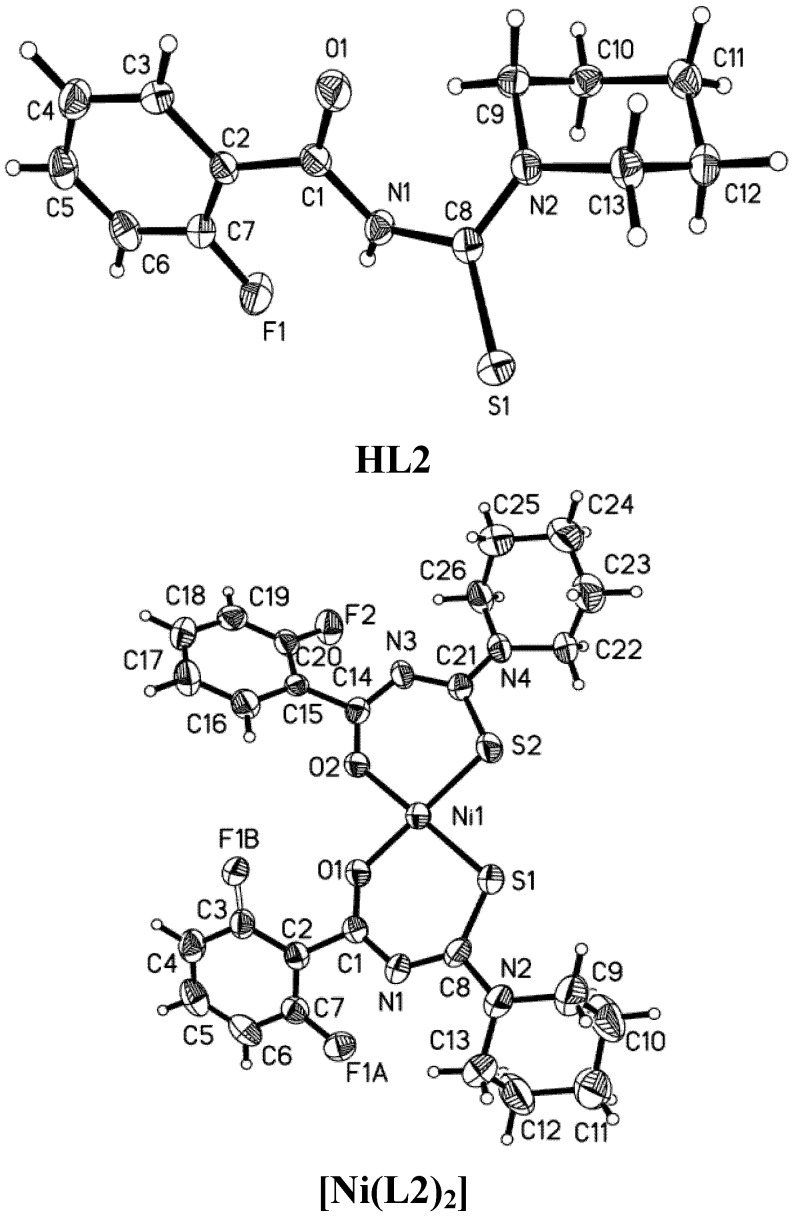
Molecular structures of HL2 and [Ni(L2)_2_], with thermal ellipsoids drawn at 40% probability.

**Figure 2:**
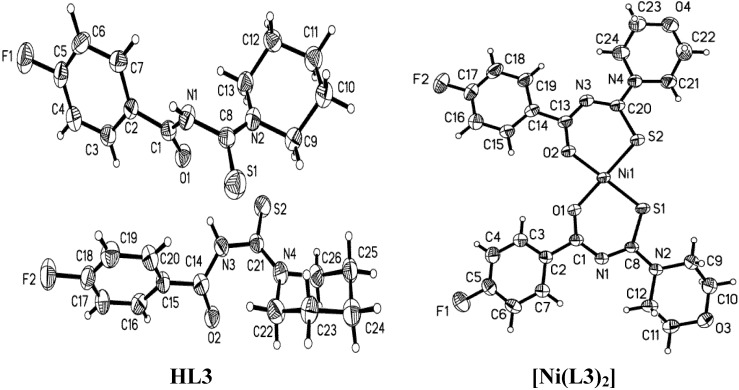
Molecular structures of HL3 and [Ni(L3)_2_], with thermal ellipsoids drawn at 40% probability.

**Figure 3:**
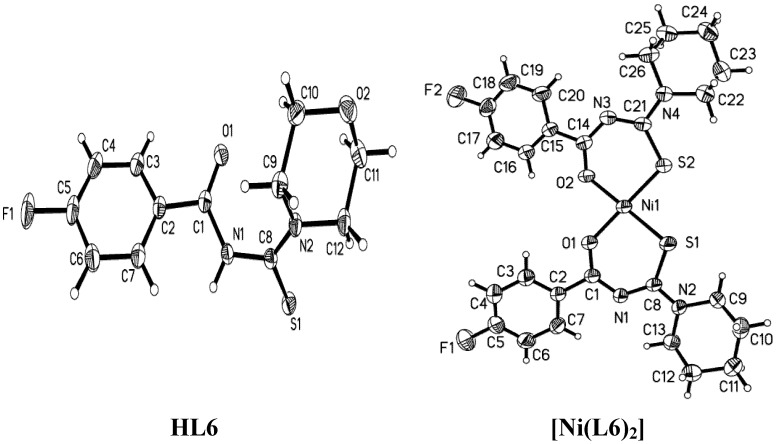
Molecular structures of HL6 and [Ni(L6)_2_] with thermal ellipsoids drawn at 40% probability.

The molecule structures of the three ligands and the corresponding complexes with Ni(II) are shown in Figure 1, Figure 2 and Figure 3, and their X-ray diffraction parameters are summarized in Table 1. Three ligands show *trans*-conformation between the C=S and C=O. No significant differences appear in the bond lengths and bond angles of the three ligands (Table 3). The amidic C(1)-N(1), thiomide N(2)-C(8) and N(1)-C(8) bonds are shorter than the C–N single bond length of 1.472(5) Ǻ. The partial double bond character of the structure is presumed as a result of the *p*-π conjugation. The length of the bond C(8)-S(1) lies between the values of C–S single and double bonds. In the crystals of HL3, two molecules have some small conformational distinctions.

**Table 1:** Summary of X-ray diffraction data.

Parameter	HL2	[Ni(L2)_2_]	HL3	[Ni(L3)_2_]	HL6	[Ni(L6)_2_]
Empirical formula	C_13_H_15_FN_2_OS	C_26_H_28_F_2_N_4_NiO_2_S_2_	C_13_H_15_FN_2_OS	C_26_H_28_F_2_N_4_NiO_2_S_2_	C_12_H_13_FN_2_O_2_S	C_24_H_24_F_2_N_4_NiO_4_S_2_
Formula weight	266.33	589.35	266.33	589.35	268.3	593.3
Crystal system	Monoclinic	Orthorhombic	Triclinic	Triclinic	Monoclinic	Triclinic
Space group	*P 21/c*	*P b c a*	*P-1*	*P-1*	*C 2/c*	*P-1*
a (Å)	11.843(3)	11.6111(17)	8.3759(15)	9.5530(16)	21.214(6)	9.3383(6)
b (Å)	13.282(3)	9.8778(17)	11.191(2)	11.890(2)	9.7391(16)	11.5273(9)
c (Å)	8.432(2)	45.925(7)	13.960(3)	12.548(2)	14.637(7)	12.7230(8)
α (°)	90	90	89.981(10)	104.350(4)	90	105.206(6)
β (°)	108.474(5)	90	89.969(10)	100.377(3)	125.91(3)	101.420(5)
γ (°)	90	90	79.699(9)	97.110(4)	90	96.112(6)
Z	4	8	4	2	8	2
Dcalc (g/cm^3^)	1.406	1.486	1.374	1.464	1.455	1.543
Radiation (MoKα) (Å)	0.71075	0.7107	0.7107	0.7107	0.71075	0.71073
μ(Mo Kα) (mm^−1^)	0.259	0.941	0.253	0.927	0.272	0.977
θ Range (°)	3.03 to 27.50	3.02 to 25.35	3.17 to 25.34	3.01 to 25.35	3.34 to 27.50	3.03 to 26.37
Reflections collected	7538	34,645	12,652	12,987	10,790	13,024
Independent reflections	2850	4799	4678	4872	2773	5220
R(int)	0.022	0.0687	0.0719	0.0292	0.0514	0.0208
Data	2850	4799	4678	4872	2773	5220
restraints	0	0	2	0	0	17
Parameters	165	344	334	335	163	334
GOF on F^2^	1.011	1.009	1.098	1.079	0.999	1.023
R1 [I > 2sigma(I)]	0.0436	0.0904	0.0838	0.0536	0.0422	0.0406
wR2 [I > 2sigma(I)]	0.1141	0.1785	0.1771	0.1242	0.1084	0.1002
R1 [all data]	0.0551	0.11	0.1575	0.0723	0.0636	0.0564
wR2 [all data]	0.1222	0.1892	0.2080	0.1353	0.1190	0.1105
Largest difference peak (e.A^−3^)	0.362 and −0.377	0.728 and −0.413	0.265 and −0.316	0.627 and −0.297	0.331 and −0.449	0.552 and −0.315

The crystal structure of [Ni(L2)_2_] shows static disorder for the fluorine atoms on one phenyl ring so the positions of atoms cannot be determined with a sufficient accuracy. All of complexes exhibit *cis*-tetracoordinate structures around nickel(II) in the crystalline molecules, which are similar to those of [Ni(L4)_2_] [32], *cis-*bis(1,1-diethyl-3-benzoylthiourea)-nickel(II) [33] and *cis-*bis(1,1-diethyl-3-(2-chlorobenzoylthiourea-O,S)-nickel(II) [34]. The slightly distorted square geometry is also found in the structure of the three complexes. The O–Ni–O, S–Ni–S and O–Ni–S angles deviate slightly from 90.0°. The conformation isomerizes, which must have happened during the coordination process. The C1–N1–C8–S1 and C14–N3–C21–S2 torsion angles (Table 2) corresponding to the *cis-*conformation are completely different from the *trans-*conformations of the ligands.

**Table 2:** Some structure parameters of the determined crystals

Distances (Å)	HL2	[Ni(L2)_2_]	HL3	[Ni(L3)_2_]	HL6	[Ni(L6)_2_]
Ni(1)-O(1)		1.859 (4)		1.864 (2)		1.861 (18)
Ni(1)-O(2)		1.846 (4)		1.866 (2)		1.864 (18)
Ni(1)-S(1)		2.130 (2)		2.140 (11)		2.145 (8)
Ni(1) -S(2)		2.147 (2)		2.139 (11)		2.142 (8)
S(1)-C(8)	1.673 (15)	1.728 (7)	1.668 (5)	1.742 (4)	1.679 (2)	1.737 (2)
S(2)-C(21)		1.723 (6)	1.666 (5)	1.731 (4)		
S(2)-C(20)						1.731 (3)
O(1)-C(1)	1.215 (19)	1.271 (7)	1.233 (5)	1.275 (4)	1.219 (2)	1.268 (3)
O(2)-C(14)		1.269 (7)	1.233 (6)	1.268 (4)		
O(2)-C(13)						1.272 (3)
N(1)-C(1)	1.378 (18)	1.319 (7)	1.358 (6)	1.312 (4)	1.392 (2)	1.321 (3)
N(3)-C(14)		1.309 (7)	1.358 (6)	1.319 (5)		
N(3)-C(13)						1.307 (3)
N(1)-C(8)	1.413 (19)	1.337 (8)	1.434 (6)	1.340 (5)	1.397 (2)	1.342 (3)
N(3)-C(21)		1.341 (8)	1.430 (6)	1.334 (5)		

The conformational disposition in the complexes must be derived from the electron delocalization along the S1–C8–N1–C1–O1 fragment. The N1–C1, N3–C14, N1–C8 and N3–C21 distances are shorter than the N1–C1 and N1–C8 distances in the ligands. The distances of N2–C8 and N4–C21 in the complexes are longer than the distance of N2–C8 in the ligands. As Table 2 shows, all N–C bond distances are between a C–N single bond (1.468 Å) and double bond (1.25 Å). The O–C and S–C bonds in the complexes are longer than those in the ligands.

### 2.2. Antifungal Activities

The average values of the minimum inhibitory concentration (MIC) of the ligands and their complexes against the fungal strains are shown in Table 3. Taking the commercial fluconazole, the ligands *N*-(piperidine-1-thioyl)benzamide (HL1), *N*-(morpholine-4-thioyl)benzamide (HL4) and their complexes, [Ni(L1)_2_] and [Ni(L4)_2_] as reference, the ligands and the complexes containing fluorine atoms had good performance inhibiting the growth of the fungi. Especially, the antifungal abilities of HL6 are equal to that of commercial fluconazole; the MIC values against the fungi *Botrytis cinerea*, *Trichoderma* spp., *Myrothecium* and *Verticillium* spp. are 8.0, 8.5, 7.0 and 8.5 μmol/L, while those of fluconazole are 7.0, 8.0, 7.5 and 7.5 μmol/L, respectively. Moreover, four ligands also have the stronger antifungal abilities than the corresponding complexes. For example, the MIC values for [Ni(L6)_2_] are 31.0 (*Botrytis cinerea*), 25.0 (*Trichoderma* spp.), 27.5 (*Myrothecium*) and 27.5 μmol/L (*Verticillium* spp.), respectively, much higher than that of HL6 (mentionedabove). The results are similar to the antibacterial activities [24]. The inhibitory abilities of the piperidinyl ligands are inferior to those of morpholine ligands. The MIC values for HL3 were 20.5 (*Botrytis cinerea*), 18.0 (*Trichoderma* spp.), 22.5 (*Myrothecium*) and 25.0 μmol/L (*Verticillium* spp.), respectively, which differ from the antibacterial activities [24]. The position effect is more apparent. The introduction of a F atom at the *para*-position of the benzoyl increased the antifungal activities notably. Contrarily, when the F atom substitutes the benzoyl *ortho*-position, the antifungal abilities decrease.

**Table 3:** The MIC of the ligands and their complexes against the studied fungi (SD: standard deviation).

Samples	*Botrytis cinerea*	*Trichoderma* spp.	*Myrothecium*	*Verticillium.* spp.
Fluconazoleole	7.0 ± 0.1	8.0 ± 0.1	7.5 ± 0.1	7.5 ± 0.1
HL1	31.0 ± 0.0	22.5 ± 0.1	32.5 ± 0.1	34.0 ± 0.0
[Ni(L1)_2_]	33.0 ± 0.0	31.5 ± 0.1	36.0 ± 0.0	38.0 ± 0.1
HL2	27.5 ± 0.0	29.0 ± 0.0	27.5 ± 0.1	28.0 ± 0.0
[Ni(L2)_2_]	31.0 ± 0.1	33.0 ± 0.1	31.0 ± 0.1	31.5 ± 0.1
HL3	20.5 ± 0.1	18.0 ± 0.1	22.5 ± 0.1	25.0 ± 0.0
[Ni(L3)_2_]	22.5 ± 0.0	31.0 ± 0.0	29.0 ± 0.1	31.0 ± 0.0
HL4	2.5 ± 0.1	2 ± 0.0	2.3 ± 0.0	0.8 ± 0.1
[Ni(L4)_2_]	29.0 ± 0.1	32.5 ± 0.1	35.5 ± 0.1	35.5 ± 0.1
HL5	31.0 ± 0.1	27.5 ± 0.0	27.5 ± 0.0	31.0 ± 0.1
[Ni(L5)_2_]	32.5 ± 0.1	31.0 ± 0.1	31.0 ± 0.1	31.5 ± 0.0
HL6	8.0 ± 0.0	8.5 ± 0.0	7.0 ± 0.1	8.5 ± 0.0
[Ni(L6)_2_]	31.0 ± 0.0	25.0 ± 0.0	27.5 ± 0.1	27.5 ± 0.0

### 2.3. Structure-Activity Relationships

On the one hand, organic molecules containing F have the good lipophilicity and thus enhanced rates of cell penetration and transport to an active site [23]. Furthermore, the fluorination adjacent to a π-system or heteroatom-containing functional group can strongly polarize the parent molecule [35]. Taking morpholine and piperidine as the donors and F atoms as the acceptors, the ligands containing F would form a D-A molecular system. The D-A model molecules will be more active in the interaction with biomolecules than the common, so the molecules should have strong antimicrobial activity.

The change of the antimicrobial activity is mostly attributed to the change of the structures in the ligands and the complexes. The change in the structures must result in the change of the charge population on the atoms of the complexes and accordingly induce the differences of antimicrobial activities. In our recent work, it was found that the lower the LUMO energy was, the more polar the C–N of the acyl was, and the higher the antibacterial activity was [29]. In the crystal structures of HL2, HL3 and HL6, the average C–N bond of the acyl measures 1.374, 1.368 and 1.392 Å, respectively. HL6 has the longest C–N bond, so the C–N polarity is the largest and the antimicrobial activity must be the strongest. Upon coordination, the C–O and C–S bonds weaken and the amide C–N bonds gradually strengthen. The average C–N bond distances of 1.319(7), 1.312(4) and 1.321(3) Å in [Ni(L2)_2_], [Ni(L3)_2_] and [Ni(L6)_2_] are shorter than those of the C–N bond of the amide, 1.378(18), 1.358(6) and 1.392(2) Å in the corresponding free ligands, HL2, HL3 and HL6. The shortened bond length of the amide C–N bond is a disadvantage for the polarity increase, so the antifungal activity of the complexes will decrease.

**Figure 4:**
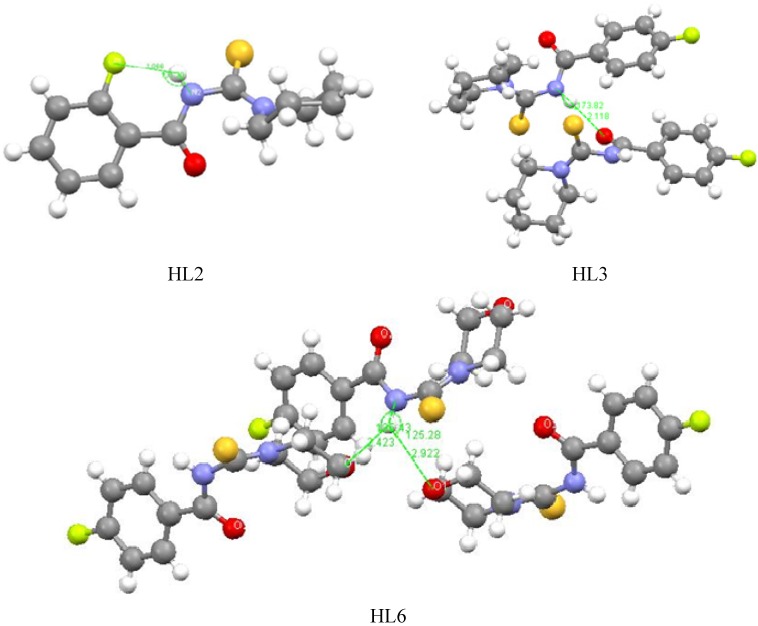
The packing diagrams of the corresponding hydrogen bonds of ligands.

For HL2, the intramolecular hydrogen bonding N–H∙∙∙F will decrease the polarity of the molecules, and is also disadvantage for the polarity increase of the C–N bond of the amide. For HL3, no intramolecular hydrogen bonding interactions exist, but, a dimer is formed in the crystals of HL3 due to the strong intermolecular N–H∙∙∙O hydrogen bonding interactions, with a H∙∙∙O distance of 2.118, N∙∙∙O distance of 2.965Ǻ, and ∠N–H∙∙∙O of 173.8°. This hydrogen bonding results in a longer C–O bond (1.233 Ǻ), which disturbs the polarity of amide C–N bond. In the crystal structure of HL6, because the O atom of morpholine replaces the carbon atom of piperidine, two weak intermolecular N–H∙∙∙O(1) interactions with H∙∙∙O distances of 2.423 and 2.922, N∙∙∙O distances of 3.408 and 3.104 Ǻ, and ∠N–H∙∙∙O, 125.3° and 135.4° are observed instead of the strong N–H∙∙∙O intermolecular hydrogen bonding interactions in HL3 (Figure 4). The weak interactions do not affect the polarity of the amide C–N bond, so HL6 has the strongest antifungal activity.

## 3. Experimental

### 3.1. Materials

Melting points were measured with Kofler melting point apparatus (Tallmadge, OH, USA) and were uncorrected. IR spectra were obtained in KBr discs using a Nicolet 170SX FT-IR spectrometer (Thermo Fisher Scientific, Shanghai, China). ^1^H-NMR (400.13 MHz) spectra were recorded in chloroform-d (CDCl_3_) solution at room temperature on an INOVA 400 instrument (VARIAN, Palo Alto, CA, USA) with TMS as internal reference. Elemental analyses were performed on a Yanaco CHNSO Corder MT-3 analyzer (PerkinElmer, Waltham, MA, USA). X-ray diffraction determination was carried out with a Rigaku Mercury CCD diffractometer (Rigaku, Japan).

### 3.2. Synthesis

**Scheme 1:**
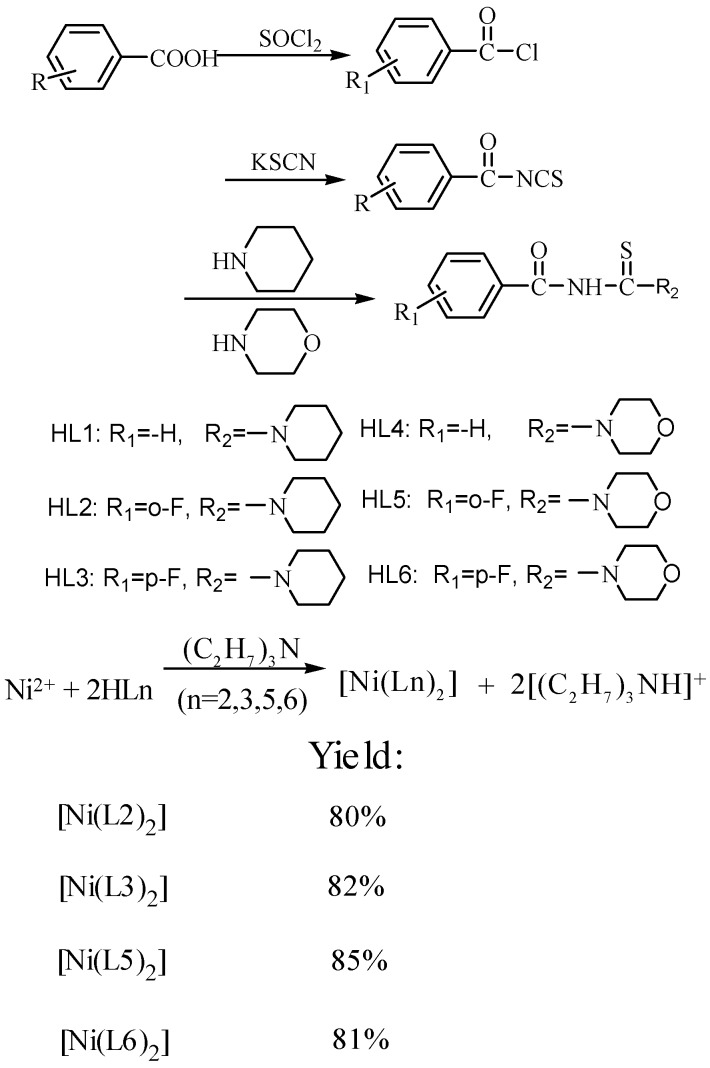
Synthetic route of the ligands and the complexes.

The synthetic route to the target ligands given in the literature [28] was used, as outlined in Scheme 1. Fluorobenzoyl chlorides were obtained by the acylation reaction of the corresponding fluorobenzoic acids with thionyl chloride.

Then, a freshly prepared acetone solution of benzoyl isothiocyanate synthesized by the condensation of fluorobenzoyl chloride with KSCN was added dropwise to a stoichiometric amount of morpholine or piperidine solution with stirring. The mixture was heated and refluxed for 1–2 h. After that, the mixture was poured on crushed ice with vigorous stirring. The isolated solids was filtered, washed with water and dried to yield the target ligands. The synthees of the complexes are also depicted in Scheme 1. The complexes were obtained by the reaction of anhydrous NiSO_4_ with the corresponding ligands (molar ratio of Ni^2+^:L = 1:2) in ethanol in the presence of triethylamine. All of ligands and complexes are air-stable enough to be used for the antimicrobial property studies.

**[Ni(L2)_2_]**: Dark green solid, m.p.:186.5–188.4 °C. Anal. Calc. for C_26_H_28_F_2_N_4_NiO_2_S_2_ (% C, H, N and S): 52.97, 4.75, 9.51 and 10.87. Found (% C, H, N and S): 52.92, 4.81, 9.46 and 10.92. FTIR (cm^−1^): 3016 (w,υ_Ph-__H_), 2938 (w, υ_CH2_), 2857 (w, υ_CH2_), 1585 (w, υ_Ph-__H_), 1526 (m, υ_Ph-__H_), 1489 (m, υ_Ph-__H_), 1414 (m, δ_CH2_), 1358 (w, υ_C-F_), 1244 (w, υ_C-O_), 1132 (w, υ_C-N_), 1020 (w, υ_C-S_), 758 (w, δ_Ph-H_). ^1^H-NMR (ppm): 7.96–7.93 (t, 2H, *J* = 6.8, 6.4 Hz), 7.38 (s, 2H), 7.14–7.10 (t, 2H, *J* = 7.2, 7.2 Hz), 7.05–7.00 (t, 2H, *J* = 9.6, 9.2 Hz), 4.06 (s, 2H), 3.96 (s, 2H), 3.96 (s, 4H), 1.68–1.61 (d, 12H, *J* = 28.8 Hz).

**[Ni(L3)_2_]**: Dark green solid, m.p.: 238.9–240.6 °C. Anal. Calc. for C_26_H_28_F_2_N_4_NiO_2_S_2_ (% C, H, N and S): 52.97, 4.75, 9.51 and 10.87. Found (% C, H, N and S): 52.95, 4.80, 9.54 and 10.80. FTIR (cm^−1^): 3030 (w, υ_Ph-__H_), 2941 (w, υ_CH2_), 2860 (w, υ_CH2_), 1601 (w, υ_Ph-__H_), 1518 (m, υ_Ph-__H_), 1483 (m, υ_Ph-__H_), 1424 (m, δ_CH2_), 1364 (w, υ_C-F_), 1244 (w, υ_C-O_), 1150 (w, υ_C-N_), 1018 (w, υ_C-S_), 856 (w,δ_Ph-H_). ^1^H-NMR (ppm): 8.10 (s, 4H), 7.05 (s, 4H), 4.08–3.98 (d, 8H, *J* = 24 Hz), 1.69 (s, 12H).

**[Ni(L5)_2_]**: Brown solid, m.p.: 229.6–230.0 °C. Anal. Calc. for C_24_H_24_F_2_N_4_NiO_4_S_2_ (% C, H, N and S): 48.57, 4.05, 9.44 and 10.79. Found (% C, H, N and S): 47.90, 4.10, 9.42 and 10.68. FTIR (cm^−1^): 3076 (w, υ_Ph-__H_), 2923 (w, υ_CH2_), 2856 (w, υ_CH2_), 1589 (w, υ_Ph-__H_), 1521 (m, υ_Ph-__H_), 1473 (m, υ_Ph-__H_), 1419 (w, δ_CH2_), 1356 (w, υ_C-F_), 1252 (w,υ_C-O_), 1110 (w,υ_C-N_), 1020 (w,υ_C-S_), 755 (w,δ_Ph-H_). ^1^H-NMR (ppm): 7.97 (d, 2H, *J* = 0.8 Hz), 7.44 (d, 1H, *J* = 2.0 Hz), 7.18–7.15 (t, 2H, *J* = 6.0, 7.2 Hz), 7.09–7.05 (t, 2H, *J* = 8.8, 8.4 Hz), 4.18–4.05 (d, 6H, *J* = 24 Hz), 3.78–3.74 (d, 6H, *J* = 16.4 Hz), 2.99–2.91 (d, 2H, *J* = 29.6 Hz), 1.61(s, 2H).

**[Ni(L6)_2_]**: Pink solid, m.p.: 302.9–303.1 °C. Anal. Calc. for C_24_H_24_F_2_N_4_NiO_4_S_2_ (% C, H, N and S): 48.57, 4.05, 9.44 and 10.79. Found (% C, H, N and S): 48.63, 3.99, 9.42 and 10.82. FTIR (cm^−1^): 3046 (w, υ_Ph-__H_), 2968 (w, υ_CH2_), 2856 (w, υ_CH2_), 1601 (w, υ_Ph-__H_), 1515 (m, υ_Ph-__H_), 1474 (m, υ_Ph-__H_), 1419 (m, δ_CH2_), 1350 (w, υ_C-F_), 1260 (w, υ_C-O_), 1150 (w, υ_C-N_), 1028 (w, υ_C-S_), 852 (w, δ_Ph-H_). ^1^H NMR (CDCl_3_) (ppm): 8.21 (s, 4H), 7.08–7.41 (t, 4H, *J* = 8.4, 8.4 Hz), 4.17–4.16 (d, 4H, *J* = 4.4 Hz), 4.05–4.04 (d, 4H, *J* = 4.4 Hz), 3.77–3.76 (d, 4H, *J* = 4.4 Hz), 2.18 (s, 2H), 1.62 (s, 2H).

### 3.3. X-ray Structure Determination

Crystals suitable for the X-ray diffraction determination were prepared by solvent evaporation method in dimethylformamide (DMF). All measurements were operated on a Rigaku Mercury CCD X-ray diffractometer [36] by using graphite monochromated MoKα radiation (λ = 0.71070 Å). Single crystals were mounted with grease at the top of a glass fiber. Cell parameters were refined on all observed reflections by using the program CrystalClear (Rigaku and MSC, Ver. 1.3, 2001) [37]. The collected data were reduced by the program CrystalClear and an absorption correction (multiscan) was applied.

The reflection data of the crystals were corrected by Lorentz and polarization effects. The crystal structures were solved by direct methods and refined by full-matrix least-squares methods (F^2^) using the SHELXTL software package [38,39]. Non-hydrogen atoms were refined anisotropically and hydrogen atoms were placed at calculated positions. The structure of [Ni(L2)_2_], involved one orientation disorder, and the molecule was refined in two positions, the main position of the atom F1A was 56.8 % and the secondary position of the atom F1B was 43.2%. The summary of the key crystallographic information and the selected bond lengths and bond angles for both crystals was listed in Table 2 and Table 3 respectively. The other information of the crystals had been deposited in the Cambridge Crystallographic Data Centre as CCDC 836,547 (HL2), CCDC 845,010 (HL3), CCDC 877,566 (HL6), 875,172 ([Ni(L2)_2_]), 878,379 ([Ni(L3)_2_]) and 882,281 ([Ni (L6)_2_]). These data can be obtained free of charge from The Cambridge Crystallographic Data Centre via www.ccdc.cam.ac.uk/data_request/cif.

### 3.4. Determination of the Minimum Inhibitory Concentration (MIC)

The MIC of the test samples was determined by a two-fold serial dilution method. We pre-cultured the studied fungi for 24 h at 28 °C in LB (Luria-Bertani) liquid medium (tryptone 10 g, yeast extract 5 g, NaCl 10 g and 500 mL of distilled water). Then, taking the same concentration of the studied fungi cultured for 24 h in LB liquid medium. Subsequently, the different concentrations of the test samples were added into the LB liquid medium containing the studied fungi and cultured for 24 h at 28 °C (dimethyl sulfoxide (DMSO) as the control and fluconazole as the reference). The culture solution (50 μL) was inoculated on the LB solid medium to observe the growth of the colony and the MIC was the highest dilution concentration of no growth of the fungi. Each concentration repeated three times for each fungus. Differences between the experimental group and the control group were analyzed by Least Significant Difference (LSD) t-test. *p* < 0.05 was considered to indicate a statistically significant difference. In this work, all results of experiments were measured up to *p* < 0.05.

## 4. Conclusions

Four fluorine-containing thiourea complexes ([Ni(L2)_2_], [Ni(L3)_2_], [Ni(L5)_2_], [Ni(L6)_2_]) have been successfully synthesized. The crystal structures of HL2, HL3, HL6 and the nickel complexes have been determined by X-ray diffraction. The [Ni(L2)_2_], [Ni(L3)_2_] and [Ni(L6)_2_] complexes have similar *cis-*tetracoordinate structures.

The antifungal activity assays show the introducing fluorine in the *para*-position of the benzoyl moiety in ligands is a very powerful and versatile tool to increase antifungal activities by changing the electronic parameters and forming a D-A system. The presence of intra- and intermolecular hydrogen bonding interactions and the coordination will be disadvantage for increasing the polarity of the amide C-N bond and this decreases the antifungal activities, so the antifungal activity of HL6 is the strongest among the studied samples, and is the same as that of the commercial fungicide fluconazole.
